# Global architecture of gestational diabetes research: density-equalizing mapping studies and gender analysis

**DOI:** 10.1186/s12937-016-0154-0

**Published:** 2016-04-04

**Authors:** Dörthe Brüggmann, Theresa Richter, Doris Klingelhöfer, Alexander Gerber, Matthias Bundschuh, Jenny Jaque, David A Groneberg

**Affiliations:** 1 Department of Obstetrics and Gynecology, Keck School of Medicine of USC, Los Angeles, California USA; 2 Department of Female Health and Preventive Medicine, Institute of Occupational Medicine, Social Medicine and Environmental Medicine, Goethe-University, Frankfurt, Germany

**Keywords:** Gestational diabetes, Diabetes mellitus, Scientometrics, Density equalizing mapping, Gender

## Abstract

**Objective:**

Gestational diabetes mellitus (GDM) is associated with substantial morbidity for mothers and their offspring. While clinical and basic research activities on this important disease grow constantly, there is no concise analysis of global architecture of GDM research. Hence, it was the objective of this study to assess the global scientific performance chronologically, geographically and in relation to existing research networks and gender distribution of publishing authors.

**Study design:**

On the basis of the New Quality and Quantity Indices in Science (NewQIS) platform, scientometric methods were combined with modern visualizing techniques such as density equalizing mapping, and the Web of Science database was used to assess GDM-related entries from 1900 to 2012.

**Results:**

Twelve thousand five hundred four GDM-related publications were identified and analyzed. The USA (4295 publications) and the UK (1354 publications) dominated the field concerning research activity, overall citations and country-specific Hirsch-Index, which quantified the impact of a country’s published research on the scientific community. Semi-qualitative indices such as country-specific citation rates ranked New Zealand and the UK at top positions. Annual collaborative publications increased steeply between the years 1990 and 2012 (71 to 1157 respectively). Subject category analysis pointed to a minor interest of public health issues in GDM research. Gender analysis in terms of publication authorship revealed a clear dominance of the male gender until 2005; then a trend towards gender equity started and the activity of female scientists grew visibly in many countries. The country-specific gender analysis revealed large differences, i.e. female scientists dominated the scientific output in the USA, whereas the majority of research was published by male authors in countries such as Japan.

**Conclusion:**

This study provides the first global sketch of GDM research architecture. While North-American and Western-European countries were dominating the GDM-related scientific landscape, a disparity exists in terms of research output between developed and low-resource countries. Since GDM is linked to considerable mortality and morbidity of mothers and their offspring and constitutes a tremendous burden for the healthcare systems in underserved countries, our findings emphasize the need to address disparities by fostering research endeavors, public health programs and collaborative efforts in these nations.

## Background

Gestational diabetes mellitus (GDM) is defined as impaired glucose tolerance with onset or first recognition during pregnancy [1]. Although GDM has been previously regarded as “benign” [2] and usually resolves shortly after delivery [3], the condition is associated with substantial morbidity for mothers and their offspring [4–9]. Specifically, fetuses exposed to an intrauterine high-glucose environment are at risk for macrosomia and adverse perinatal outcomes such as injury or asphyxia during birth, infant respiratory distress syndrome, hypoglycemia or hyperbilirubinemia [10]. They are also prone to develop diabetes or obesity later in life; epigenetic changes might be responsible for this trans-generational transmission of diabetes [11]. Pregnancies of affected patients are often complicated by gestational hypertension or preeclampsia; rates are also increased for delivery by cesarean section or significant trauma during vaginal delivery [12]. Maternal long-term morbidities include GDM recurrence in a subsequent gestation and the development of type 2 diabetes after 5 to 10 years [13].

Glucose intolerance during pregnancy is common in almost every population around the globe [14]. Genetic, social and environmental factors predispose women to GDM, particularly females of Hispanic, African, Native American, Asian, and Pacific Island ancestry [15, 16]. In the United States of America (USA), approximately 200,000 new GDM cases are diagnosed each year translating to an annual economic burden of over $600 million [17, 18]. GDM rates are estimated to be 10 % in the USA, 5 % in the UK, and 2-6 % in other European countries [19]. These numbers are expected to further increase due to growing obesity and sedentary lifestyle [20]. GDM is also an aggravating problem in low-resource countries: In 2015, the majority of 21 million live births affected by hyperglycemia in pregnancy were reported in low-and middle-income countries. South-East Asia was leading the field with a prevalence of 24.2 %; in comparison, rates in Africa were reported up to 11 % [21, 22]. In these countries, GDM poses a tremendous burden: The condition leads to significant perinatal mortality, e.g. as shown in Kenya by a perinatal mortality rate of 254 in 1000 [23]. Commonly, diagnosis and treatment of the condition are challenging due to insufficient financial resources and infrastructure as well as climate and geography impacting the access and availability of care. Also, the compliance of patients is hampered by lack of education, religious beliefs, superstitions and fear of stigma [24].

Controversy still remains regarding the diagnosis and treatment of GDM [16]. Scientific evidence is largely insufficient of which screening criteria are the most reliable to diagnose GDM, when and who to screen, and whether particular screening practices of GDM would improve maternal and perinatal outcomes significantly [25, 26]. Screening practices are not uniform worldwide leading to a frequent under-diagnosis of GDM, incorrect assessment of local prevalences as well as an under-management of affected women [15]. Hence, GDM constitutes a major public health concern. Uniform management criteria are crucial for the global establishment of successful primary and secondary preventive strategies carried out in the framework of international collaborations. In view of the burden GDM creates worldwide, detailed knowledge on the global research architecture of GDM is required for (1) individual scholarship, (2) planning future scientific and public health initiatives according to identified shortcomings, and (3) objective performance assessment in the field to supply decision makers with information concerning funding strategies. This is a challenging task due to the vast number of related scientific publications available. Therefore, scientometric methods enable researchers to conduct a focused, systematic and reliable analysis of journal articles regarding their content and citations to describe trends in institutions of origin and dissemination of published data. Specifically, this ‘New Quality and Quantity Indices in Science’ (NewQIS) project [27, 28] aimed to assess the scientific output related to GDM regarding quantitative and qualitative aspects, geographical and chronological developments, existing research networks and gender analysis in a standardized way using an in-depth study protocol combing scientometric tools and modern visualization techniques including density equalizing mapping [29].

## Methods

### NewQIS platform

We employed the previously validated New Quality and Quantity Indices in Science (NewQIS) platform to identify all research published on GDM since 1900 [27, 28]. Our search covered original articles but also publications such as meeting abstracts and reviews and included clinical, basic science and translational research conducted on animals or humans. In 2009, NewQIS was established as part of an international study project [27, 28]. It encompasses both scientometric and novel visualization techniques such as density equalizing mapping projections (DEMP) to illustrate research activity by anamorphic maps for the purpose of benchmarking processes aiming to compare qualitative and quantitative performance metrics of biomedical research [27, 28]. Since NewQIS started, numerous studies have been performed in the fields of obstetrics [30], public health and health policy development [31–35], or internal medicine [36, 37].

### Data source

Similar to previous studies, we used the Web of Science database (WoS, Thomson Scientific) for data collection. This resource enabled us to conduct a unique citation analysis in addition to the assessment of publication activity [38, 39]. No research was performed involving human subjects or animals hence it was not required to obtain IRB approval for this study.

### Search strategy

NewQIS studies are based on unique search terms that encompass the disease of interest. Hence, we generated the GDM-specific search term “Topic = diabetes AND (gestation* OR “pregnancy induced” OR “pregnancy associated” OR maternal)” to approximate the overall number of published items. The term was applied in a “Topic” search identifying the search term in the title, abstract and author’s keywords. The analyzed timeframe for GDM research covered the years between 1900 (01–01) and 2012 (31–12). Results from 2013 onwards were not considered due to incomplete data acquisition at the time the study was performed.

### Data analysis and categorization

According to the previously described workflow [27, 28], we downloaded and saved all publications identified by our search term in a Plain text format using the download tool provided by the WoS. Then, an interim database was created by collecting all metadata related to the items. Based on these metadata, GDM-related publications were analyzed and categorized with respect to publication date, document type, country of origin, language, source title, subject categories, authors, and participating institutions. Also, citation information was retrieved for each publication; the average number of citations per item (citation rate) and the modified Hirsch-Index (h-Index) were calculated as semi-qualitative variables. We related the citation rate and h-Index, which represents the impact of one author’s research output on the scientific community, to the GDM-specific publication activity of single countries constituting a country-specific citation rate and “modified” h-Index.

After transfer of the downloaded and unedited WoS plain text data files to excel charts, the findings were illustrated in diagrams and visualized by DEMPs. The current DEMPs were based on the algorithm of Gastner and Newman [29]. Here, the territories of the different countries publishing on GDM were resized in proportion to our selected variables. We used this technique to draw a sketch of the global GDM research activities concerning the distribution of country-specific numbers of published items and average citation rates [29]. All investigated countries were categorized as high-income, upper-middle and lower-middle income countries according to the definition of the World Bank (http://data.worldbank.org/country/).

### Gender analysis

In order to determine gender aspects of global GDM research, we calculated the proportion of male to female authors (reflected by the male to female ratio, m:f ratio) based on all authors’ names listed in the publications. We conducted a manual search (utilizing websites, corresponding addresses and social networks) if first names were not gender-specific or quoted as initials. In particular, Asian names were difficult to assign to a specific gender. To avoid imprecision, only countries were considered with a minimum of 500 authors who published more than 700 items in total and whose author’s gender could clearly be determined in over 50 % of names. This methodology has been previously described in earlier research [29].

### Analysis of cooperations

To analyze scientific co-operations between different countries, author affiliations stated on the identified GDM-related publications were analyzed as previously described [38, 40]. Only countries that participated in five or more collaborations were included in the analysis. If at least two authors conducting research in two different countries contributed to one GDM publication, this relationship was defined as collaboration. We termed collaborations involving two countries as bilateral and collaborations involving three countries as trilateral cooperations. To visualize the productivity of these co-operations for each pair of countries a vector was calculated, which was proportional in line width and shade of grey to the number of GDM-related collaborations [38, 40]. We also calculated the country-specific percentage of collaborative publications among all published items in high-income, upper-middle and lower-middle income countries (Table 1) and assessed differences between these groups by Student’s *t*-test. A *p*-*value* <0.05 was regarded as statistically significant.

**Table 1:** Publication output in relation to population size

Country	Number of publications	Collaborative publications	Joint publications in %	Population size	P/P Index	Country type
USA	4295	702	16.3	314,112,078	13.7	HI
UK	1354	459	33.9	63,700,300	21.3	HI
Australia	635	172	27.0	22,728,254	27.9	HI
Canada	584	162	27.7	34,754,312	16.8	HI
Italy	550	177	32.2	59,539,717	9.2	HI
Germany	536	161	30.0	80,425,823	6,7	HI
France	497	129	26.0	65,639,975	7.6	HI
Sweden	430	149	34.7	9,519,374	45.2	HI
Israel	367	105	28.6	7,910,500	46.4	HI
Spain	366	92	25.1	46,773,655	7.8	HI
China	231	65	28.1	1,350,695,000	0.2	UMI
Brazil	268	38	14.2	202,401,584	1.3	UMI
South Africa	38	13	34.2	52,341,625	0.7	UMI
Serbia	20	8	40.00	7,199,077	2.8	UMI
Jamaica	13	8	61.5	2,707,805	4.9	UMI
Thailand	27	9	33.3	67,164,130	0.4	UMI
Algeria	17	11	64.7	37,439,427	0.5	UMI
Tunisia	22	7	31.8	10,777,500	2.0	UMI
India	142	48	33.8	1,263,589,639	0.1	LMI
Egypt	25	15	60.0	856,009,902	0.02	LMI
Sri Lanka	13	6	46.2	20,328,000	0.6	LMI

We related the total numbers of publications to the population number of high-income (HI), upper-middle (UMI) and lower-middle income (LMI) countries (publications/population-index(P/P Index): number of publications/population in millions). Also, the number of collaborative publications and the percentage of these among the total publications are listed

### Analysis of publication output related to the number of inhabitants

The research output of the ten most productive high-income, eight upper-middle income and three lower-middle income countries was related to the number of inhabitants aiming to analyze a country’s scientific productivity in the context of manpower. The following index was calculated: publications/population-index: number of publications/population in millions. We obtained the 2012 population statistics from the World Bank (http://data.worldbank.org/country/).

## Results

### General parameters

**Fig. 1:**
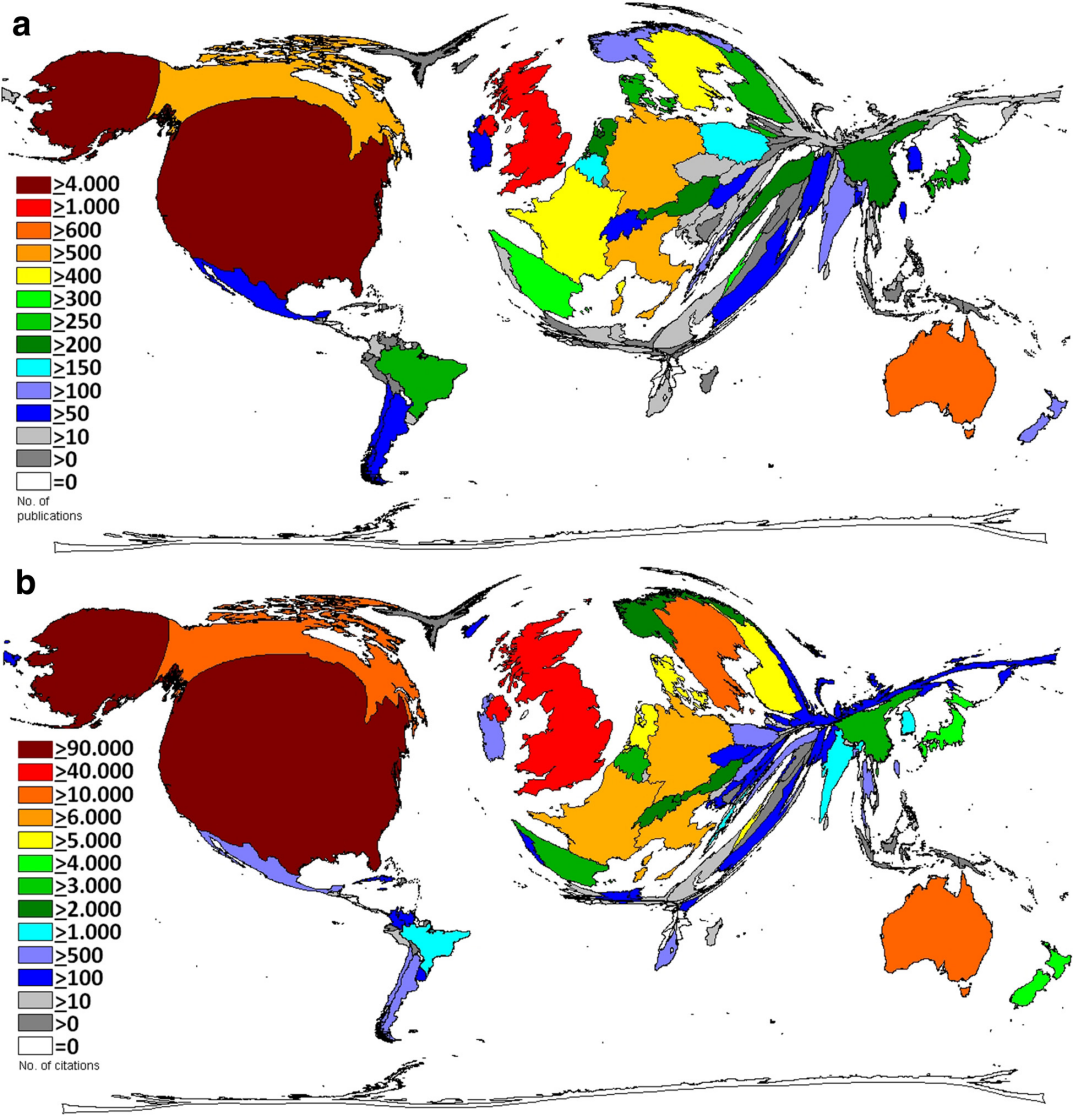
Density-equalizing map of global research activity related to gestational diabetes mellitus in the timeframe between 1900 and 2012. Colors and territorial sizes indicate numbers of publications (**a**) as well as numbers of citations per country (**b**)

In total, 12,504 publications related to GDM research were identified; 95.22 % of these were authored in English. The USA was the most productive county with 4,295 publications (p: publications), followed by the United Kingdom (UK, p: 1,354), Australia (p: 635), Canada (p: 584), Italy (p: 550), Germany (p: 536), France (p: 497), Sweden (p: 430), Israel (p: 367) and Spain (p: 366). This distribution of the global research productivity is depicted in the DEMP-analysis (Fig. 1a): Here, the USA constitutes the scientific center with the clear majority of publications (p: 4,295), followed by Western Europe. In contrast, major parts of Asia including Russia and China, Africa and South America appear minimized due to their extremely low scientific output, e.g. South Africa (p: 38, Chile p: 90 and India p: 142).

### Country citations

When we analyzed the total citation activity, the USA was the leading nation with 93,567 citations, followed by the UK with 44,443 citations, and Australia with 12,341 citations. Canada had 11,136 citations and Sweden 10,719 citations, followed by France (7,186 citations), Germany (6,830 citations) and Italy (6,621 citations). The Netherlands was ranked 9th with 5,823 citations and Finland 10th with 5,580 citations.

DEMP illustrating the citation analysis (Fig. 1b) demonstrates a similar picture to the DEMP analysis of total publication activity (Fig. 1a). Some minor exceptions were noted, i.e. Sweden occupied a higher proportion of the world map. Overall, the Scandinavian countries held a more pronounced position when citations were analyzed compared to publication activity.

### Country specific h-Index

**Fig. 2:**
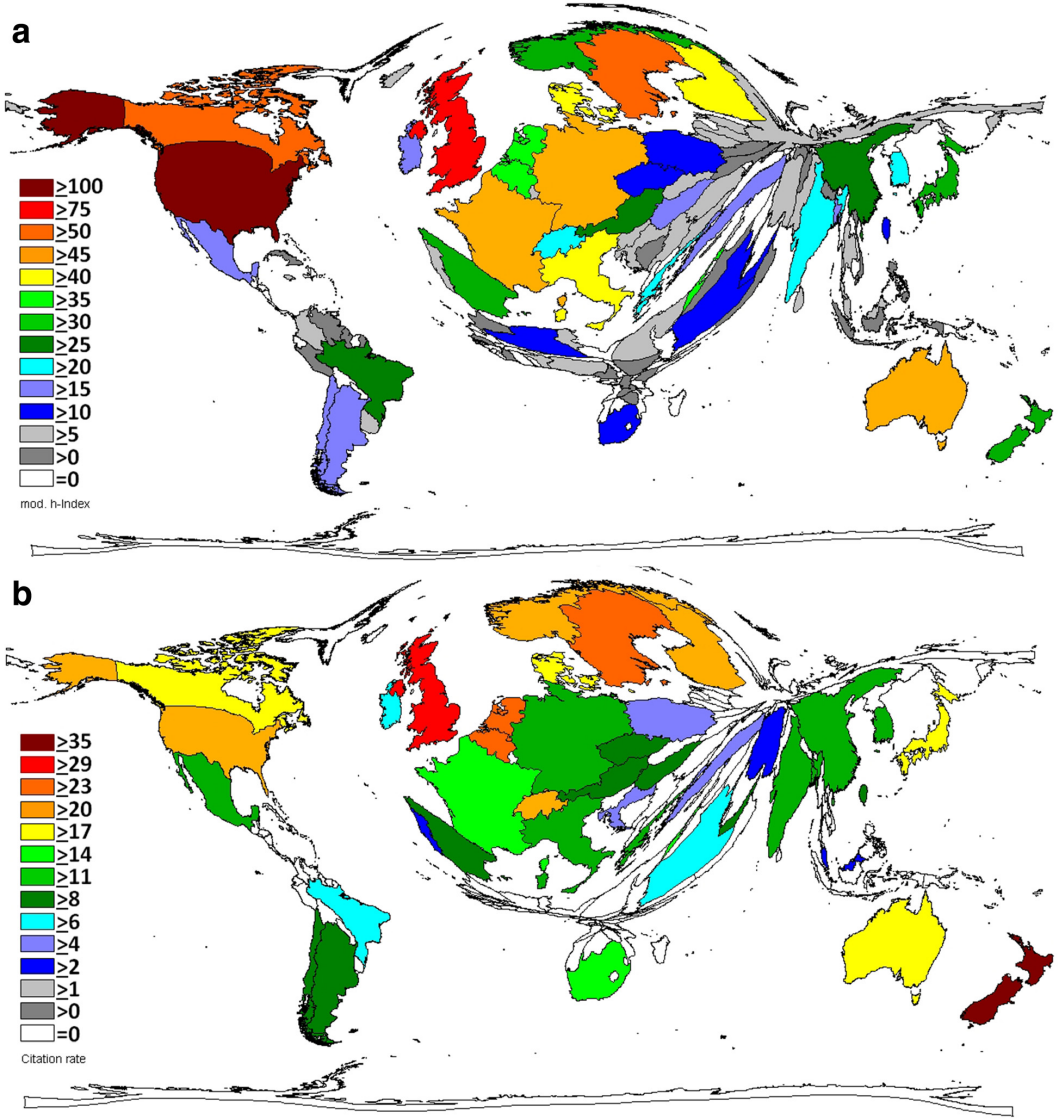
Density-equalizing map projections of semi-qualitative variables assessed for GDM-related research. **a** Country-specific Hirsch-index. **b** Citation rate (citations per publication of a country, with a threshold of 30 publications per country)

The calculation of the country specific h-Index demonstrated a leading position of the USA with 125 GDM-related publications being cited at least 125 times. The USA was followed by the UK with a country specific h-Index of 97, Canada (h-Index: 55), Sweden (h-Index: 53), Australia (h-Index: 48), Germany (h-Index: 47) and France (h-Index: 45). Italy had a country-specific h-Index of 42 and Finland and Denmark both 41. Again, countries from Asia, Africa, Eastern Europe, South and Central America occupy only a minimized area on the DEMP map corresponding with their extremely low modified h-Indices (Fig. 2a).

### Country citations rates

The country citation rate (CR) is a semi-qualitative measure (citations per publication of a country, with a threshold of 30 publications per country) in contrast to absolute parameters such as the total publication and total citation counts. In this analysis of GDM-specific research, New Zealand was ranked first with a CR of 36.26 (4,859 citations and 134 publications). Again, the UK occupied the second place with a CR of 32.82, followed by Sweden (CR 24.93), Belgium (CR 23.78), Netherlands (CR 23.77), Switzerland (CR 22.48), and Finland (CR 21.81). The USA was only ranked 8th with a CR 21.79 followed by Norway (CR 20.55) and Australia (CR 19.43) (Fig. 2b).

### Gender analysis

**Fig. 3:**
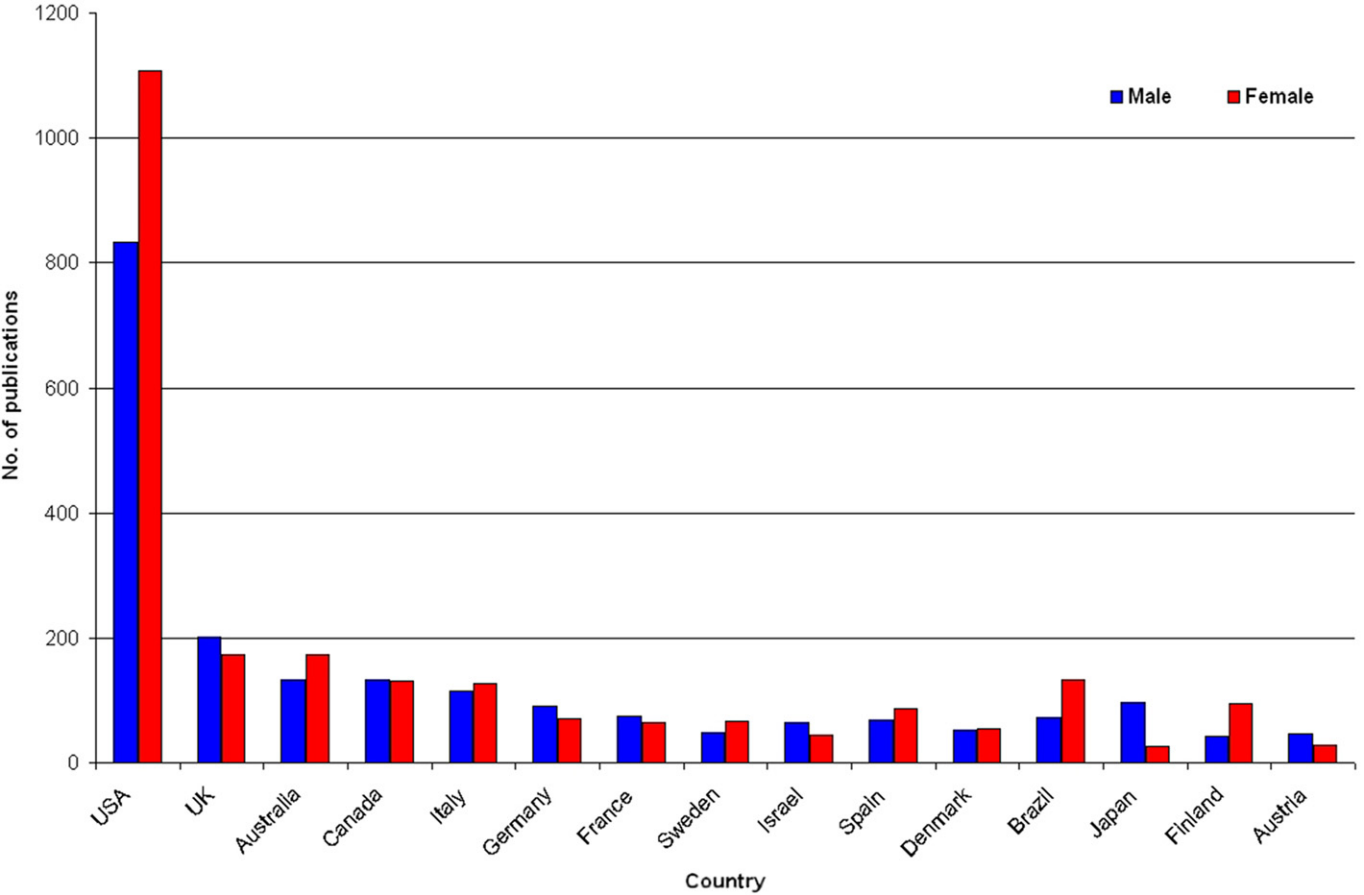
Gender analysis of the 15 most publishing countries. The proportion of female and male authors as depicted here was assessed based on the authors’ names listed in the publications

When the gender analysis of authors active in GDM research was performed, an increase in the percentage of female authorship occurred from 2005 onwards. In the assessment of the 15 most productive countries, large differences were present as demonstrated by 1107 female versus 832 male scientists who authored all US-American publications (m:f ratio of 0.8). In Canada, the gender distribution was almost equal as seen by 134 female versus 131 male authors (m:f ratio of 1.0). In striking contrast, 97 male versus 27 female scientists (m:f ratio of 3.6) were identified for Japanese authors of GDM-related publications (Fig. 3). Overall, we identified males representing the largest proportion of scientists in five of the 15 evaluated countries including the UK (201 males versus 174 females; m:f ratio of 1.2), Germany (90 males versus 70 females; m:f ratio of 1.3), France (75 males versus 65 females; m:f ratio of 1.2), Japan and Austria (46 males versus 29 females; m:f ratio of 1.6). In the USA, Australia (134 males versus 173 females; m:f ratio of 0.8), Italy (114 males versus 127 females; m:f ratio of 0.9), Sweden (49 males versus 66 females; m:f ratio of 0.7), Spain (68 males versus 86 females; m:f ratio of 0.8), Brasil (72 males versus 134 females; m:f ratio of 0.5) and Finland (42 males versus 95 females; m:f ratio of 0.4), a higher percentage of women were active in publishing GDM-specific research. An equal distribution of female to male authors was seen for Canada and Denmark (52 males versus 55 females; m:f ratio of 1).

### Collaborative articles

**Fig. 4:**
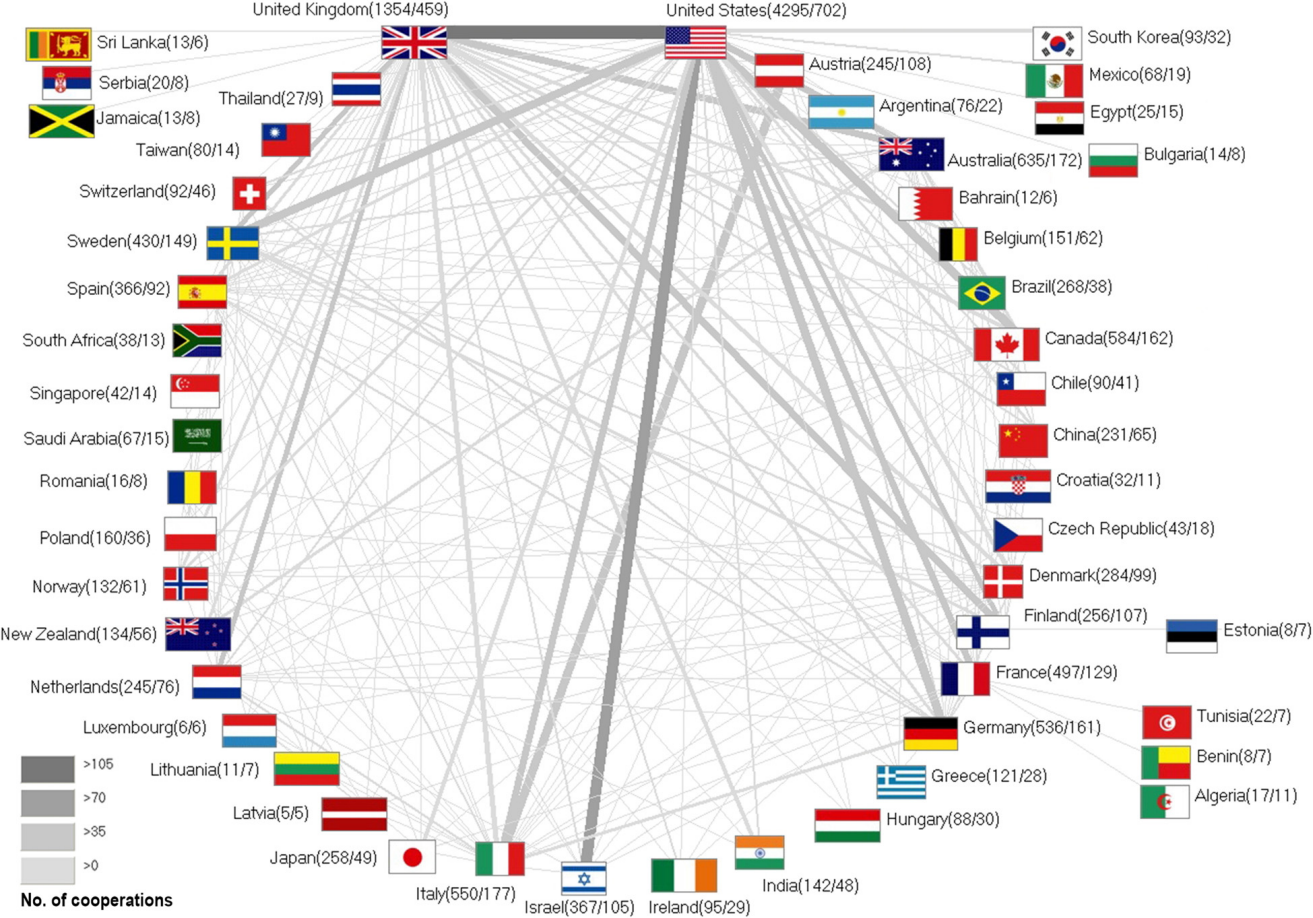
International cooperations. This spider chart exemplifies the extent of cooperations between the all investigated countries. Greyscale and bar thickness indicate intensity of collaborations. Figures beside the country names represent the number of publications/number of collaboration articles

In total, 1,261 of all publications were a result of international collaborations between two or more countries (10,1 % of all publications). The annual number of collaborative publications increased steeply between the years 1990 and 2012 (71 to 1157 respectively). Bilateral cooperations issued 1,261 publications and were the most common type, followed by trilateral collaborations (195 publications). Cooperations between four countries authored 41 items and collaborative efforts between five countries created 21 publications. 15 publications were issued by authors working in six different countries. Again, the USA dominated this analysis with 702 collaborative articles (16.3 % of all US-American publications). Most productive cooperations were established between the USA and the UK (110 joint publications), followed by the USA and Israel (83 publications), the USA and Canada (69 publications) and the USA and Sweden (66 publications). The UK was part of 459 cooperations (33.9 % of all UK publications), while Italy - as the third most active collaborating country - issued 177 publications (32.2 % of all Italian publications). A spider chart exemplifies the extent of cooperations between all investigated countries (Fig. 4).

We also assessed the percentage of collaborative publications among all country-specific publications as shown in Table 1. On average, high-income countries published 29.5 %, upper-middle income countries 38.5 % and lower-middle income countries 46.7 % items in a joint effort. We found significant differences between the percentage of collaborative items among all country-specific publications when we compared high-income to upper-middle income as well as low-income countries: The high-income countries issued less publications in joint efforts than authors from upper-middle income (*p*: *0.042*) or low-income countries (*p*: *0.001*).

### Subject area analysis

**Fig. 5:**
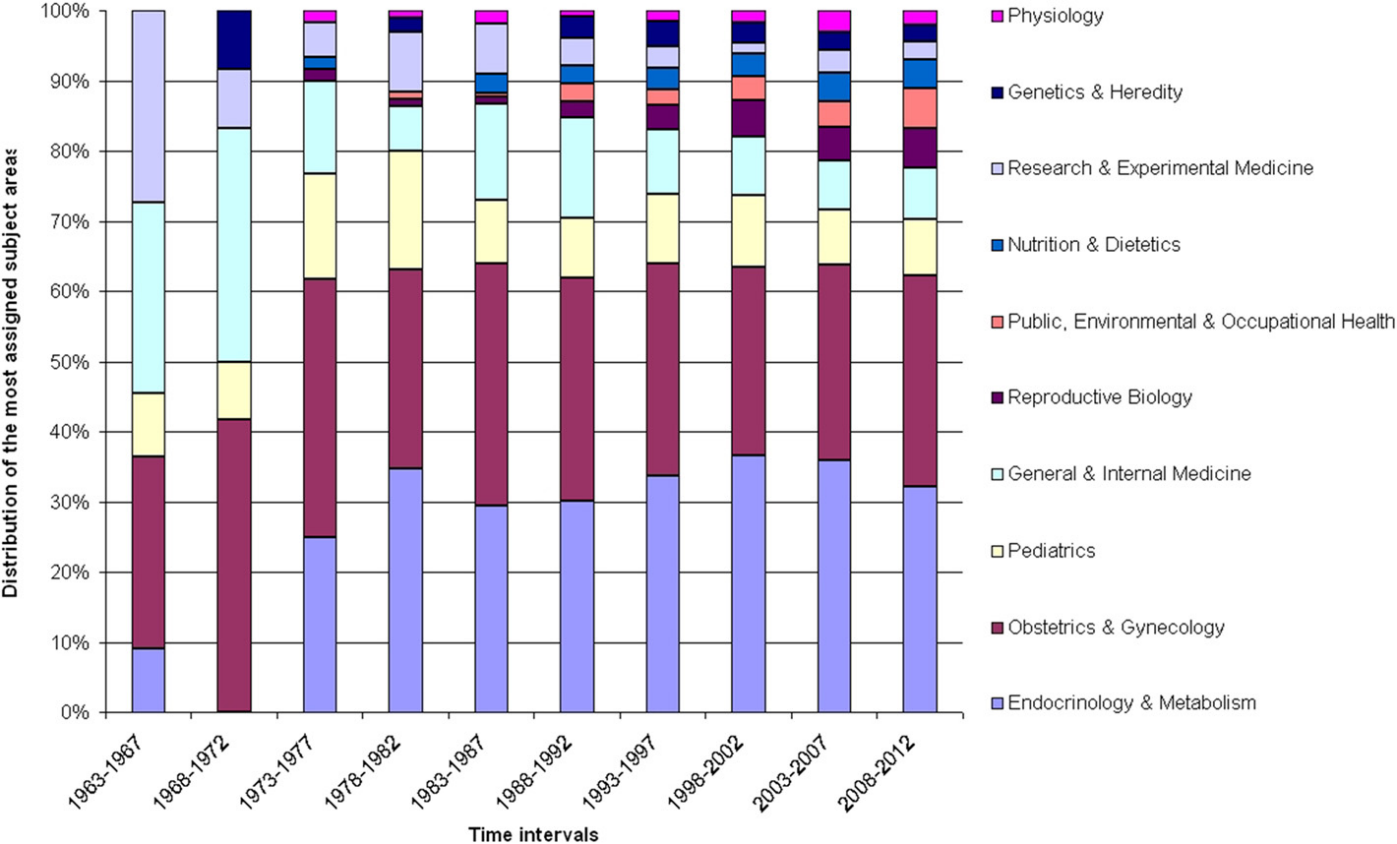
Subject areas. Relative proportions of the 10 most assigned subject areas illustrated in 5-year intervals between 1900 and 2012

The subject categories of all GDM-related articles were analyzed in five-year intervals from 1963 to 2012 with regard to their percentage proportion in order to gain insights into the field’s activity and scientific priorities (Fig. 5). Overall, we documented a percentage increase in the subject area ‘Endocrinology & Metabolism’. In the period of 1963–1967, only a small proportion was attributed to this subject area. This grew over time, and from 1978 onwards this category dominated the research together with ‘Obstetrics and Gynecology’ (OB/GYN), which was the second most applied subject category. The percentage in the category “General and Internal Medicine” decreased in relative numbers after 1972. Further, the field diversified after 1973 and articles were published in numerous new categories such as “Physiology”, “Genetics and Heredity”, “Nutrition and Dietetics” and “Public, Environmental and Occupational Health”.

### Analysis of publication output related to the number of inhabitants

When GDM-specific research output was related to population size, we documented the following ranking: Israel was leading the analysis with 46.4 GDM-specific publications per citizen, followed by Sweden (45.2 GDM-specific publications per citizen), Australia (27.9 GDM-specific publications per citizen), UK (21.3 GDM-specific publications per citizen) and Canada (16.8 GDM-specific publications per citizen). The USA (13.7 GDM-specific publications per citizen) dropped to position six among the 10 most productive high-income countries. Very low indices were found for countries with large population sizes such as China (0.2 GDM-specific publications per citizen) and India (0.1 GDM-specific publications per citizen) (Table 1).

## Discussion

This study is the first detailed analysis of the global GDM research architecture. It combines established scientometric and density equalizing mapping tools [29] and focuses on gender aspects of this important female health issue. We identified 12,504 publications related to GDM research, which have been authored since 1900. The scientific output was dominated by the industrialized North-American, Western European nations as well as Israel; these nations commonly established scientific collaborations leading to numerous publication of outstanding quality. In contrast, many developing and underdeveloped countries did not participate in research or collaborative efforts dedicated to GDM.

Before going into detail, the following methodological strengths and limitations need to be discussed. For our analysis, we employed the NewQIS platform, which constitutes a novel study tool that was specifically designed for standardized, objective and reliable scientometric analyses of research productivity based on a validated protocol [27, 28]. Benefits of using this sophisticated platform include the efficient evaluation of the scientific progress chronologically and geographically, the visualization of the results in expressive global maps as well as the incorporation of unique investigations such as the institution network analysis. As data source, we preferred the WoS to the PubMed database because the publications listed in the WoS are attached with a broader range of easily accessible bibliographic data (e.g. subject categories). Also, the WoS supports the Journal Citation Reports, which are a WoS-specific feature to extract detailed citation information for single index entries [41]. Hereby, we could assess semi-qualitative variables, i.e. the country specific h-indices, enabling us to evaluate the research productivity in a multifaceted way. Therefore, we believe that the WoS is the most suited database to address the presented research question. However, we are well aware that entering the present search term in the PubMed search function would have lead to a different set of identified publications because differing scientific journals are indexed in this particular database. A methodological issue we identified in this study is a language bias. 95.22 % of GDM-related publications were authored in English. Since the WoS has a clear preference for English journals and many national, non-English written journals are not enlisted by the WoS, non-English items might be underrepresented in our analysis. Therefore, we have to assume a bias towards all anglophone countries such as the USA, UK and Australia that needs to be taken into account when interpreting the country-specific GDM research productivity. However, we consider this particular language bias as limited since all high quality research of non-English speaking countries is commonly published in English [42]. Also, 95 % of cited and 80 % of published items related to a specific topic are indexed by the WoS and therefore would have been incorporated in our analysis [43]. The third limitation is related to the complexity of the gender analysis in the presented study. Since it is not possible to discriminate between sexes for gender-neutral or abbreviated names, i.e. for the Chinese authors, we had to exclude publications originating from China from this specific part of our analysis. However, China did not belong to the top 15 most productive countries, so the problem can be considered as minor. When assessing the gender of the publishing scientists in a country-specific manner, differences became evident: Whereas the USA seemed to contribute well to the gender mainstreaming with a dominance of female authors, other countries such as Japan do not seem to address this issue with evident success, i.e. by the establishment of specific programs supporting women. Promoting women is important in a surgical specialty such as OB/GYN: Presently, female physicians are increasingly entering surgical professions [44, 45] although surgery is still a male-dominated area with women representing only 10–20 % of the workforce [45–47]. In contrast to this development, the percentage of female medical school faculty in surgical fields remains well below the proportion of men [45, 48]. This indicates a major disparity and disadvantage for females to become promoted into higher academic ranks. Therefore, specific programs need to be established that address discrimination in medicine and science. As shown for GDM, Brazil - among other countries (e.g. the USA, Australia, Italy) - serves as an impressive example supporting the participation of female scientists in economy, science and technology as numerous gender benchmarking studies demonstrated [49]. We want to point out that other nations like Japan should feel encouraged to follow this nation’s lead.

Overall, we identified the USA as the leading country in GDM research: It was the most productive nation and was involved in the majority of collaborative efforts. US-American publications gained the highest number of citations and had the highest h-index. Countries that participate in close collaborations with the USA such as Canada seem to clearly benefit from this cooperation as documented by Canada’s outstanding output and quality in research. When the global GDM research activity is compared to other fields of medicine, it can be stated that the dominance of the USA is a common phenomenon, e.g. as shown by Groneberg-Kloft [33]. The authors assessed the overall publication output in the biomedical field related to 21 organ systems. Here, the USA issued 1,893,800 of 5,527,558 publications in 50 years and showed the highest productivity. We believe the US-American dominance in the medical-scientific field points to the excellent research conditions and funding situation in this country. For GDM, we documented the following ranking regarding publication productivity: After the USA the UK was ranked second, Australia third, Canada 4th, Italy 5th, Germany 6th, France 7th, Sweden 8th, Israel 9th and Spain 10th. We related these results to other bibliometric studies in the field of obstetrics, and a similar picture emerged. For smoking and pregnancy, 10,043 publications were assessed and the majority of items were authored by the USA, the UK and Canada followed by other Northern European nations [30]. In our study, it also became apparent that Japan does not seem to focus on GDM research with the same emphasis as put on other diseases. This finding may be linked to the relatively low prevalence of GDM in Japan, which is documented at 2.9 % [50].

We deduced the need to generate a systematic sketch of the global GDM research architecture from the fact that reliable data on GDM prevalences as well as on related perinatal mortality or morbidity are scarce in many parts of the world. Since diagnostic criteria to identify GDM are not standardized globally, it is difficult to quantify and compare reported epidemiological data to draw meaningful conclusions [24]. A recent study aimed to investigate the prevalence and geographical patterns of GDM in low-and middle-income countries [51]: The local rates of GDM varied, no reliable geographical patterns were confirmed, and prevalences were estimated between 0.4 % and 24.3 % based on the criteria applied (e.g. 1.50–15.5 % based on the American Diabetes Association criteria, 20.8 % based on the Australian Diabetes in Pregnancy Society criteria, and 0.4–24.3 % based on the World Health Organization criteria) [51]. Overall, Vietnam, India and Cuba had the highest GDM rates, and data from Africa were particularly limited, and records on maternal mortality due to GDM were sparse [51]. It was concluded that the existing data are insufficient to build a clear picture of the burden of GDM in low-and middle-income countries and that further research is needed [51]. In addition to these recently published results, our density equalizing mapping projections also demonstrated that large parts of the world-particularly affected by GDM-are characterized by a low scientific productivity and need to increase systematic research activities to meet these obvious shortcomings. In this context, it cannot be recommended that low-resource countries invest in expensive basic science research, i.e. new pharmacologic approaches in GDM management, but should be empowered to implement local public health programs and standardized screening protocols. Unfortunately, the area of public health does not merit strong interest as demonstrated by our current subject area analysis. While there was a generally increased activity in public health research from 1963 to 2012, the overall interest in this area remained low in comparison to other subject categories. Hence, the present study may be used as a starting point to convince supra-national funding agencies to allocate resources to this extremely important area of medicine that targets both pregnant women and their offspring.

Hunt and Schuller reviewed studies that examine the prevalence of GDM and related trends with regard to different populations. In this context, differences in the local prevalence might correlate to GDM research activity: In low-risk populations, such as in Sweden, the prevalence in population-based studies is lower than 2 %-even when universal testing is offered [52]. This may lead to the assumption that GDM research is not particularly focused upon in Sweden, which is a Northern European country with a relatively low population of about 9.5 million inhabitants and relatively small active research community [53]. In our analysis, exactly the opposite was true since Sweden belonged to the most active and dedicated countries – particularly when related to manpower - that also published high quality research as demonstrated by a citation rate with 24.93 citations per GDM publication. We hypothesize that these findings are based on the unique scientific infrastructure established in this country: Data can be acquired from numerous large epidemiological databases, e.g. the Swedish Birth register, enabling researchers to conduct high quality, highly cited key epidemiologic studies. Countries with high GDM prevalences and growing obesity should have an interest to conduct research in the field aiming to lower the burden of this condition for the female inhabitants and their offspring. As shown in our study, this correlation is particularly true for the USA. However, countries located on the Asian (e.g. India and China) and African continents - where the majority of 21 million live births affected by gestational hyperglycemia are found and linked to significant mortality and morbidity [22]- were not identified as particularly active countries in GDM research. This finding may result from the personal, institutional and national challenges connected to conducting research in low-resource settings [54]. Here, planning of research endeavors and resource allocation are often hindered due to an unfavorable political climate; research is not a national priority [54]. Particularly, funding is limited and largely dependent on non-governmental sources [54]. Also, common problems in these settings include lack of an appropriate infrastructure and state-of–the art research facilities [55]. Researchers themselves face difficult conditions such as poor earnings, lack of recognition and subpar training opportunities, which often lead to their migration abroad [56].

Although our study documented a higher percentage of publications from upper- and lower-middle income countries to be joint works in comparison to high-income countries (38.5 % and 46.7 % versus 29.5 % respectively), we deduce that collaborative efforts between countries of different economic capabilities have to be further increased to improve the research productivity of low-resource nations. Challenges to establish these fruitful collaborations are manifold and include cultural differences, a diverging perspective regarding the conduct of research and unrealistic assessment of the local research capacity and resources [54]. Collaboration partners have to be willing to overcome these knowing that joint efforts in the global research arena allow the participants to tap into global knowledge and provide ample opportunities for personal development, successful research funding, and facility improvement.

Numerous studies validated that hyperglycemia during pregnancy translates into increased perinatal morbidity for mother and child; these complications can be avoided and lessened by the better detection and management of the condition [57]. Hence, the worldwide establishment of uniform screening and management criteria is crucial for future successes. Further, understanding the racial and ethnic disparities in GDM diagnosis, management, and outcomes is also a substantial step towards implementation of meaningful policies, clinical decision making and patient counseling leading to an improvement of maternal and child health. In this context, we advocate to address country-specific challenges and to focus on close-knit networks and shared public health efforts. Strengthened collaborations allow the exchange of epidemiological data, ideas and resources between industrialized and low-resource nations and represent an opportunity-and almost an ethical responsibility-to strengthen research endeavors and to lower the burden of GDM in underserved countries.

## Conclusions

By analyzing quantitative and semi-qualitative aspects of the scientific output related to GDM, we provided an objective assessment of the research performance in the field. Also, the first sketch of the global research architecture was presented. Clearly, the USA dominated most parameters. However, other countries that usually also dominate the top five most productive countries such a Japan, did not exhibit an extremely high publication activity in GDM compared to other fields of medicine. Many low-resource countries that are affected by a considerable burden by GDM are underrepresented in the global map of GDM research. To lower the public health burden attached to GDM and to tackle the existing disparities, dedicated research needs to be tailored to apparent needs and fostered by strengthening and funding collaborative efforts between industrialized and underserved countries.

## Abbreviations

CR: citation rate
DEMP: density equalizing map projections
GDM: gestational diabetes mellitus
NewQIS: new quality and quantity indices in science
OBS/GYN: obstetrics/gynecology
UK: United Kingdom
USA: United States of America
WoS: web of science
